# Joint contribution of body mass index and psychological distress to short and long sickness absence among young and early midlife public sector employees: a register-linked follow-up study

**DOI:** 10.1093/eurpub/ckag092

**Published:** 2026-06-10

**Authors:** Mari-Liis Kalima, Tea Lallukka, Eira Roos, Jatta Valkonen, Anna C Svärd

**Affiliations:** Department of Public Health, Faculty of Medicine, University of Helsinki, Helsinki, Finland; Department of Public Health, Faculty of Medicine, University of Helsinki, Helsinki, Finland; Department of Public Health, Faculty of Medicine, University of Helsinki, Helsinki, Finland; Department of Public Health, Faculty of Medicine, University of Helsinki, Helsinki, Finland; Department of Public Health, Faculty of Medicine, University of Helsinki, Helsinki, Finland

## Abstract

Overweight/obesity and psychological distress often co-exist and are associated with sickness absence (SA). The objective of this study was to examine joint contribution of overweight/obesity and psychological distress on short and long SA periods, and to explore potential synergistic interactions between these factors. The Helsinki Health Study survey collected in 2017 among 19–39-year-old Finnish municipal employees was linked to employer’s SA registers (1–7 days for short and 8+ days for long SA periods), including participants consenting to linkage (*n* = 3966, 80% women). The mean follow-up time was 2.1 years. We calculated body mass index (BMI) from weight and height and evaluated psychological distress using the emotional wellbeing subscale of RAND-36 health-related quality of life survey. Rate ratios (RRs) and their 95% confidence intervals (CIs) were calculated for SA periods using negative binomial regression models. Interaction between overweight/obesity and psychological distress was examined using the Synergy Index (S). Most participants (85%) had at least one short and over one-fourth (29%) one long SA period. Overweight/obesity (42%) and psychological distress (23%) jointly contributed to short (RR, 1.62; 95% CI, 1.44–1.82) and long (RR, 2.48; 95% CI, 2.09–2.95) SA periods, compared to those with healthy weight and no psychological distress. The interaction between overweight/obesity and psychological distress was additive for short (*S* = 1.11), and synergistic for long SA periods (*S* = 1.40). Sociodemographic factors, working conditions, and health behaviors only slightly attenuated these associations. It is important to consider co-occurrence of overweight/obesity and psychological distress in prevention of SA.

## Introduction

Overweight/obesity [1] and common mental disorders are major public health issues [2].

In Europe, almost 60% of the adult population live with overweight/obesity [3] and the numbers are still rising in several Western countries [4]. In Finland, overweight affects 53% and obesity 20% of Finnish adults aged under 40, and prevalence of overweight and obesity has been rising, especially among working-age adults [5]. Overweight/obesity increases the risk of several chronic diseases, and it is associated with sickness absence (SA) [6, 7] and disability pension [8].

Psychological distress can be defined as symptoms of mental health problems in everyday life. Higher levels of psychological distress can reflect common mental disorders [9, 10]. Among Finnish adults aged under 40, psychological distress increased significantly between 2018 and 2024 and now affects one in three young and early midlife adults [11]. Recent global events such as the COVID-19 pandemic, the climate crisis, and rising food and energy costs are likely to have contributed to the mental health of young adults in particular [12]. Previous research also suggest that higher levels of psychological distress are associated with SA [13, 14]. In Finland, young employees’ overall SA increased in 2023 [15], common mental disorders being the leading cause [16].

Overweight and psychological distress are both associated with adverse health outcomes [2, 3] and may interact to amplify these effects [17]. These conditions are more prevalent among individuals with a lower socioeconomic position and they frequently co-occur [17], yet evidence on their combined impact on self-reported work ability and sickness absence remains limited. The compounded association of these prevalent health determinants [3, 11] may be substantial for young employees who have long working careers ahead of them. Our previous study showed that overweight/obesity and common mental disorders are jointly associated with disability retirement among midlife and older employees. In addition, common mental disorders contributed synergistically to the association between overweight/obesity and disability retirement due to musculoskeletal diseases among women [18]. To the best of our knowledge, joint contribution of overweight/obesity and psychological distress to SA have only been examined in one study [19]. This study consisted of Dutch employees (mean age 35 years) and showed that the combination of overweight/obesity and psychological distress was associated with long-term SA (>2 weeks) among women, but no synergistic interaction was found. Sociodemographic factors, working conditions, and health behaviors are associated with overweight/obesity, psychological distress, and SA. They may therefore contribute to the observed associations [20–24].

The main aim of this study was to examine the joint contribution of overweight/obesity and psychological distress to both short and long SA periods among young and early midlife Finnish employees compared to no psychological distress and healthy weight.

## Methods

### Participants

**Figure 1.**
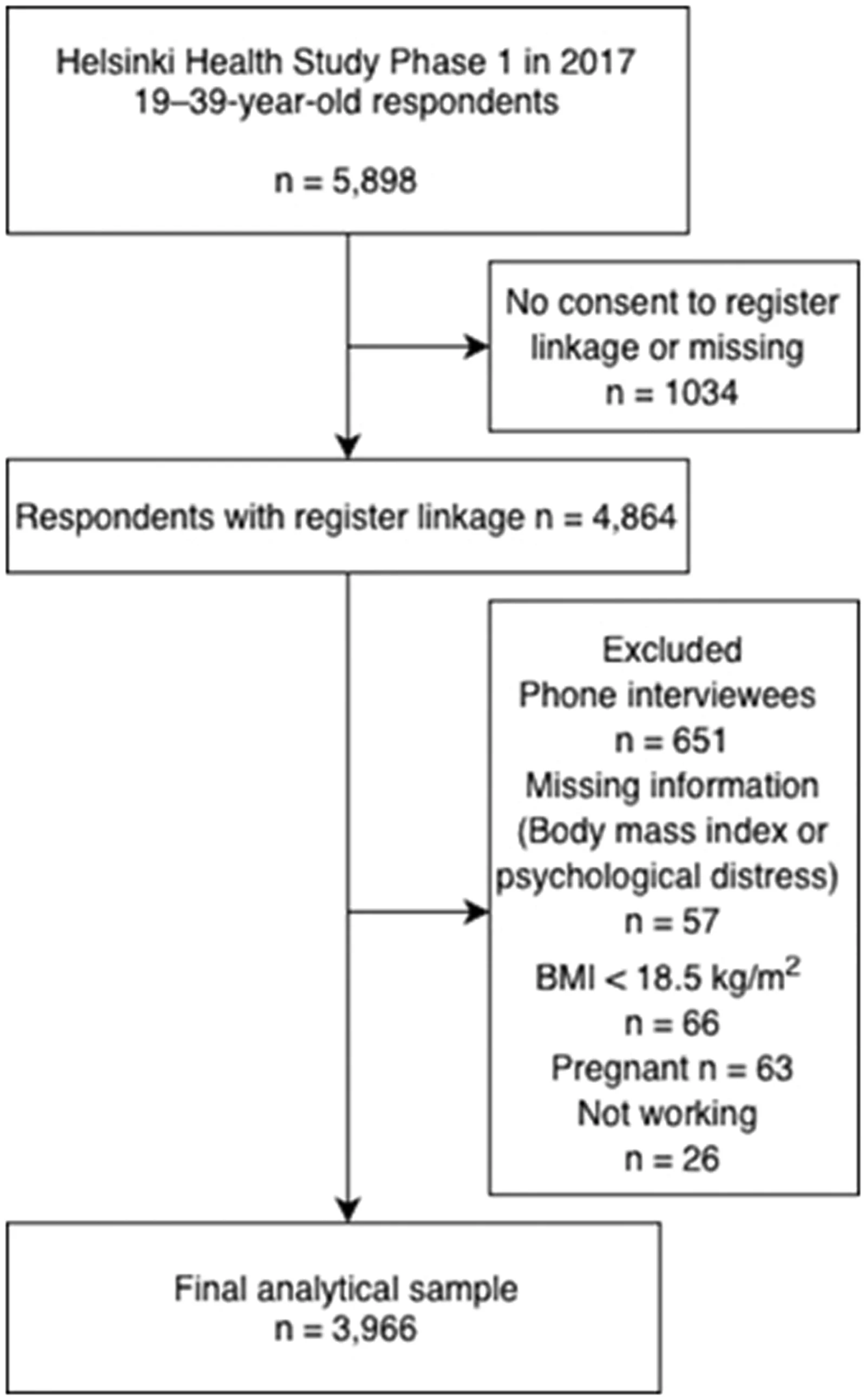
Flow chart of study participants. Young and early midlife employees of City of Helsinki. Created using draw.io (diagrams.net).

This prospective cohort study is part of the Helsinki Health Study, an ongoing cohort study that examines health and well-being, and their social and work-related determinants among employees of the City of Helsinki, Finland [25]. The City of Helsinki is the largest employer in Finland and had ∼38 500 employees in 2024. They represent hundreds of occupations, with most working in education, social, and healthcare services. Data collection in the Helsinki Health Study Phase 1 in 2017, was via online and postal questionnaires from 19 to 39-year-old employees who had had at least a 50% employment contract for at least 4 months before the start of data collection (*n* = 11 459) (Fig. 1). A shorter telephone interview was conducted with those who did not respond to the online or postal questionnaire (*n* = 787). The overall response rate was 51.5% (*n* = 5898). The questionnaire data were linked with the employer’s personnel register data on the SA for those who consented to the linkage (*n* = 4864, 82%). The majority of respondents were women (80%), consistent with the gender division of the target population and municipal workers in Finland [25].

The respondents who declined the register-linkage (*n* = 1034) were excluded. Phone-interviewed respondents were excluded due to missing variables of interest (*n* = 651). We also excluded participants with sick leave for longer than 6 months or who were receiving a disability pension (*n* = 26), those who were pregnant (*n* = 63), those who had no SA follow-up data (*n* = 31), or those with underweight (body mass index [BMI] <18.5 kg/m^2^) (*n* = 66). We excluded those with underweight due to feasibility and because they were so few in number (ca. 1%). Finally, we excluded participants with missing information on their weight (*n* = 43), psychological distress (*n* = 14), or education (*n* = 4). The final analytical sample consisted of 3966 respondents, of whom 3164 were women and 802 men.

### Measures

#### Body mass index

We calculated BMI (kg/m^2^) using weight and height reported in the survey. We used the World Health Organization (WHO) criteria to categorize participants as having either a healthy weight (BMI 18.5–24.9 kg/m^2^) or overweight/obesity (BMI ≥25 kg/m^2^) as the number (*n* = 616) of participants with obesity (BMI ≥30 kg/m^2^) was small.

#### Psychological distress

Psychological distress was assessed using the emotional wellbeing subscale of RAND-36, which is widely used to assess health-related quality of life [10, 26]. The subscale consists of five questions on mental wellbeing during the last 4 weeks. It reflects symptoms of anxiety, depression, and positive mood (“Have you been a very nervous person?,” “Have you felt so down in the dumps that nothing could cheer you up?,” “Have you felt calm and peaceful?,” “Have you felt downhearted and blue?,” and “Have you been a happy person?”). Symptoms were reported on a 6-level scale ranging from “none of the time” to “all the time.” A transformed score ranging from 0 to 100 was calculated. The emotional wellbeing subscale is identical in both the SF-36 and RAND-36 [10], although some subscales have minor coding differences. Both are well-validated screening tools and their results are comparable [10, 27]. MHI-5 is derived from SF-36, which requires a fee for use. RAND-36 and SF-36 correspond to each other and MHI-5 is comparable to RAND-36 emotional wellbeing subscale. We chose to dichotomize the participants using a cut-off score of 60, because previous studies on MHI-5 have found 60 points or less to indicate moderate or severe psychological distress [9, 27]. Additionally, a study on MHI-5 and long-term SA suggested 60 points as the best dichotomization cut-off [28]. These cut-off points on MHI-5 have been found to be optimal for determining case prevalence and minimal misclassification [9].

#### Joint exposure groups

The exposure groups were formed on the basis of the participants’ BMI and psychological distress in Phase 1 as follows: participants with (i) healthy weight and no psychological distress (BMI 18.5–24.9 kg/m^2^, emotional wellbeing score >60), (ii) healthy weight and psychological distress (BMI 18.5–24.9 kg/m^2^, emotional wellbeing score <60), (iii) overweight/obesity and no psychological distress (BMI ≥25 kg/m^2^, emotional wellbeing score >60), and (iv) overweight/obesity and psychological distress (BMI ≥25 kg/m^2^, emotional wellbeing score <60).

### Sickness absence

Participants’ SA data were retrieved from the personnel register of the City of Helsinki. The SA follow-up started on the day after receiving the questionnaire and continued until the end of participants’ contract or until 31 March 2020 (beginning of the COVID-19 pandemic in Finland, which affected employer’s SA policies), whichever came first. The mean follow-up time was 2.1 years. We examined SA periods according to their length [1–7 days (yes vs no) and 8+ days (yes vs no)]. SA periods of 1–7 days and 8+ days were analyzed as separate, non-mutually exclusive outcomes. At the time of the follow-up, City of Helsinki employees could stay home for up to 7 days due to sickness with their supervisor’s approval, without needing a medical certificate. Thus, 1–7 days of SA were considered self-certified and short periods, which are more common among young than older employees [29], while 8+ days measured long SA periods.

### Covariates

Factors potentially contributing to the association of overweight/obesity and psychological distress with SA were considered [21] and derived from the questionnaire. We classified gender as women and men [30], it corresponded 100% to juridical sex available in the registers. We treated age as a continuous variable. We dichotomized marital status into married/cohabiting and others (single, divorced, or widowed) [20]. We classified education into three groups: upper or lower secondary school, bachelor’s degree, and master’s degree or higher [21]. For work status, we dichotomized participants into those working full-/part-time and those who were temporarily studying or on parental leave. We included participants on parental leave and students, as they likely returned to work after their leave, evident from their follow-up time in the employer’s personnel register. The physical strenuousness of work was dichotomized into non-strenuous (very/rather light) and strenuous (very/rather strenuous) [31]. Alcohol consumption was dichotomized into those consuming less than weekly or not at all and those consuming alcohol weekly or more, given that weekly drinking is associated with SA [22]. Smoking status was classified as non-smoking (including ex-smokers) and smoking (current daily and occasional) [23]. Physical activity was calculated from estimates of average weekly hours of leisure-time physical activity (LTPA) and commuting activity across four intensity grades. We multiplied weekly hours by metabolic equivalent task (MET) values and then summed the values. We dichotomized MET hours so that <20 MET hours per week indicated low LTPA and more than that indicated medium or high LTPA [24, 32]. For covariates with missing values, we applied a conservative approach by coding the missing responses as specific reference categories [single (*n* = 8), having physically non-strenuous work (*n* = 34), non-weekly alcohol users (*n* = 151), non-smoking (*n* = 25), and having high levels of LTPA (*n* = 43)]. This approach minimizes the potential overestimation of associations between exposures and outcome.

### Statistical analyses

**Figure 2.**
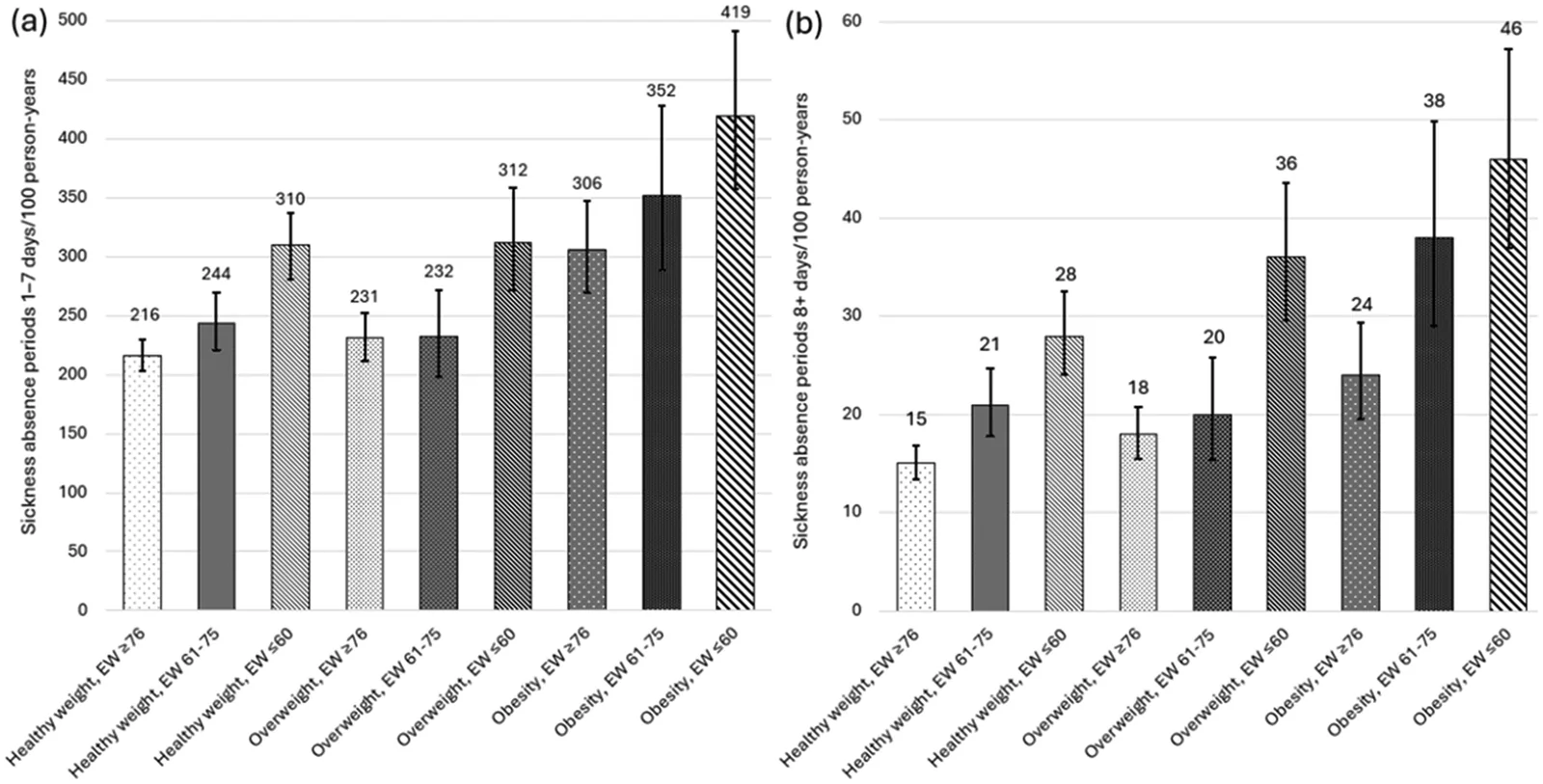
Sickness absence periods of overweight/obesity (body mass index ≥25 kg/m^2^) and psychological distress (emotional well-being score ≤60) of (a) 1–7 days and (b) 8+ days among young and early midlife employees of City of Helsinki, Finland (*n* = 3966) during mean follow-up of 2.1 years at Phase 1, 2017 (per 100 person-years with 95% confidence intervals, CIs).

First, we used cross-tabulations to describe participants’ Phase 1 characteristics according to whether they had short (1–7 days) or long (8+ days) SA periods during the follow-up, reporting *n* (%), measures of variation, and *P*-values (Table 1). Second, we calculated the incidence and their 95% confidence intervals (CIs) for short and long SA periods per 100 person-years using negative binomial regression for the four exposure groups (Fig. 2).

**Table 1.** Characteristics of young and early midlife employees of City of Helsinki at Phase 1 in 2017, by subsequent sickness absence (SA) periods of 1–7 days and 8+ days during a mean follow-up of 2.1 years

	All	SA period of 1–7 days			SA period of 8+ days		
	*n* (%)	No	Yes	*P*-value^a^	No	Yes	*P*-value^a^
		*n* (%)	*n* (%)		*n* (%)	*n* (%)	
Total SA, *n* (%)	3966 (100)	601 (15)	3365 (85)		2810 (71)	1156 (29)	
Gender							
Women	3164 (80)	457 (14)	2707 (86)	.013	2210 (70)	954 (30)	.006
Men	802 (20)	144 (18)	658 (82)		600 (75)	202 (25)	
Age							
19–29	1235 (31)	185 (15)	1050 (85)	.005	861 (70)	374 (30)	.553
30–34	1355 (34)	237 (17)	1118 (83)		970 (72)	385 (28)	
35–39	1376 (35)	179 (13)	1197 (87)		979 (71)	397 (29)	
Marital status							
Married/cohabiting	2646 (67)	405 (15)	2241 (85)	.705	1885 (71)	761 (29)	.447
Other	1320 (33)	196 (15)	1124 (85)		925 (70)	395 (30)	
Education							
Upper secondary school	1297 (33)	159 (12)	1138 (88)	<.001	835 (64)	462 (36)	<.001
Bachelor’s degree	1493 (38)	222 (15)	1271 (85)		1047 (70)	446 (30)	
Master’s degree or higher	1176 (30)	220 (19)	956 (81)		928 (79)	248 (21)	
Work status							
Working	3573 (90)	435 (12)	3138 (88)	<.001	2476 (69)	1097 (31)	<.001
Studying or on parental leave	393 (10)	166 (42)	227 (58)		334 (85)	59 (15)	
Physically strenuous work							
Non-strenuous	2642 (67)	414 (16)	2228 (84)	.200	1944 (74)	698 (26)	<.001
Strenuous	1324 (33)	187 (14)	1137 (86)		866 (65)	458 (35)	
Alcohol consumption							
Occasional or never	2843 (72)	398 (14)	2445 (86)	<.001	1980 (70)	863 (30)	.008
Weekly	1123 (28)	203 (18)	920 (82)		830 (74)	293 (26)	
Smoking							
Former or never	3021 (76)	497 (16)	2524 (84)	<.001	2181 (72)	840 (28)	<.001
Current	945 (24)	104 (11)	841 (89)		629 (67)	316 (33)	
Leisure-time physical activity^b^							
Medium or high	3382 (85)	525 (15)	2857 (85)	.118	2419 (72)	963 (28)	0.025
Low	584 (15)	76 (13)	508 (87)		391 (67)	193 (33)	
BMI^c^							
Healthy weight	2311 (58)	367 (16)	1944 (84)	.072	1712 (74)	599 (26)	<.001
Overweight	1075 (27)	164 (15)	911 (85)		758 (71)	317 (29)	
Obesity	580 (15)	70 (12)	510 (88)		340 (57)	240 (41)	
Psychological distress^d^							
None	3045 (77)	483 (16)	2562 (84)	0.024	2241 (74)	804 (26)	<.001
Psychological distress	921 (23)	118 (13)	803 (87)		569 (62)	352 (38)	

a *P*-values from Chi-squared tests.

b Leisure-time and commuting physical activity (LTPA): <20 metabolic equivalent (MET) hours per week indicates low LTPA and at least 20 MET hours per week indicates medium or high LTPA.

c Body mass index (BMI): normal weight 18.5–24.9 kg/m^2^, overweight 25–29.9 kg/m^2^, and obesity ≥30 kg/m^2^ (individuals with BMI <18.5 were excluded due to low numbers, 1.1%).

d Psychological distress: score of 60 points or less indicates psychological distress and score of over 60 points indicates no psychological distress. Measured with the RAND-36 questionnaire.

Third, we calculated rate ratios (RRs) and their 95% CIs for short and long SA by the exposure groups using negative binomial regression (Table 2). Participants with healthy weight and no psychological distress served as the reference group. Model 1 was adjusted for age and gender, and Model 2 was additionally adjusted for marital status, education, work status, and physical strenuousness of work. Model 3 was adjusted for age, gender, and health behaviors (alcohol consumption, smoking, and physical activity). The distribution of number of SA days showed overdispersion; thus, negative binomial regression was used instead of, for instance, Poisson regression to provide a better model fit for the count data. The natural logarithm of follow-up time was included as an offset to account for varying follow-up lengths. As we found no statistically significant gender interactions in the associations between the exposure groups and SA (*P = .*170 for short SA periods; *P = .*405 for long SA periods), we analyzed women and men together, adjusting for gender.

**Table 2.** Joint associations of overweight/obesity (body mass index ≥25 kg/m^2^) and psychological distress (emotional wellbeing score ≤60) with subsequent sickness absence (SA) periods of 1–7 days and 8+ days among young and early midlife employees of City of Helsinki at Phase 1, 2017 (rate ratios, RRs and their 95% confidence intervals, CIs)

SA periods	Exposure group	*n* (%)	Model 1^a^		Model 2^b^		Model 3^c^	
			RR	95% CI	RR	95% CI	RR	95% CI
1–7 days	Psychological distress/overweight							
	Neither	1815 (46)	1.00		1.00		1.00	
	Psychological distress only	496 (13)	1.35	1.21–1.51	1.29	1.15–1.44	1.28	1.14–1.43
	Overweight only	1230 (31)	1.21	1.11–1.31	1.12	1.03–1.22	1.11	1.02–1.21
	Both	425 (11)	1.62	1.44–1.82	1.45	1.29–1.64	1.42	1.26–1.60
8+ days	Psychological distress/overweight							
	Neither	1815 (46)	1.00		1.00		1.00	
	Psychological distress only	496 (13)	1.67	1.40–1.99	1.58	1.32–1.89	1.57	1.31–1.88
	Overweight only	1230 (31)	1.36	1.18–1.56	1.22	1.06–1.40	1.20	1.05–1.39
	Both	425 (11)	2.48	2.09–2.95	2.18	1.83–2.60	2.13	1.78–2.55

a Model 1: Adjusted for age and gender.

b Model 2: Adjusted for age, gender, marital status, education, work status, and physical strenuousness of work.

c Model 3: Adjusted for age, gender, alcohol consumption, smoking, and leisure-time and commuting physical activity.

As a supplementary analysis, we categorized participants into those with healthy weight/overweight (18.5–29.9 kg/m^2^) and those with obesity (≥30 kg/m^2^) (Table S1). We also conducted a supplementary analysis with the commonly used cut-off for SA periods; 1–14 days and 15+ days (Table S2).

Finally, as an outcome variable, we calculated the synergy index (S) to examine the synergistic interaction between overweight/obesity and psychological distress, using the following equation: S = (RR participants with overweight/obesity and psychological distress − 1)/[(RR participants with healthy weight and psychological distress − 1) + (RR participants with overweight/obesity without psychological distress − 1)] [33]. We used the RRs from Model 1 and fully adjusted RRs from Model 3 for the calculations. The results were similar and we chose to present the age- and gender-adjusted results. A synergy index of >1 suggests an additive or synergistic interaction. We used IBM SPSS Statistics 27 for all the analyses.

### Ethical aspects

Appropriate ethical aspects have been followed in all phases of the study, according to the Declaration of Helsinki. Participation in the study was voluntary and full confidentiality was guaranteed. An informed consent to link survey and register data were obtained from each participant. The Helsinki Health Study protocol was approved by the Ethics Committee of the Faculty of Medicine, University of Helsinki and the City of Helsinki health and personnel authorities. The Faculty of Medicine gave a positive statement for the study protocol (14 February 2017) and because the study is observational, no other ethical approval was required. The City of Helsinki updated their approval for the Helsinki Health Study protocol on 21 June 2023. https://www.helsinki.fi/en/researchgroups/helsinki-health-study

## Results

The majority of the participants had at least one SA period during the follow-up; most participants (85%) had at least one short SA period and over one-fourth (29%) had at least one long SA period (Table 1). Crosstabulation in Table 1 provides a descriptive overview of the characteristics of the study population. Almost one-fourth of the participants (23%) reported psychological distress and two-fifths (42%) had overweight/obesity. Overall, the SA periods were more common among women and among participants who had a lower education level, temporarily were not working, or who smoked.

The rate of the age- and gender-adjusted both short and long SA periods per 100 person-years during the follow-up was the highest among the participants with simultaneous overweight/obesity and psychological distress (Fig. 2). Additionally, the rate of SA periods was higher among the participants with overweight/obesity or psychological distress than among those with a healthy weight and no psychological distress.

Table 2 presents the RRs and their 95% CIs for short and long SA periods among participants according to exposure groups. The participants with overweight/obesity and psychological distress had the highest age- and gender-adjusted RRs for both short SA periods (RR, 1.62; 95% CI, 1.44–1.82) and long SA periods (RR, 2.48; 95% CI, 2.09–2.95) in comparison to the participants with a healthy weight and no psychological distress. Adjustment for marital status, education, work status, the physical strenuousness of work (Model 2), and further adjustment for health behaviors (Model 3) only slightly attenuated the associations. The interaction of overweight/obesity and psychological distress was additive for short SA periods (*S* = 1.11) and synergistic for long SA periods (*S* = 1.40).

In the supplementary analysis for which we used BMI ≥30 kg/m^2^ as a cut-off point (Table S1), the RRs were somewhat higher for both short and long SA periods. Participants with obesity and psychological distress had higher rates of short SA periods (RR 1.58; 95% CI, 1.34–1.87) than those with overweight/obesity and psychological distress (RR 1.42; 95% CI, 1.26–1.60). The results regarding long SA periods followed a similar pattern: the participants with obesity and psychological distress had higher rates of SA (RR 2.26; 95% CI, 1.78–2.87) than the participants with overweight/obesity and psychological distress (RR 2.13; 95% CI, 1.78–2.55). We also conducted the analyses with a SA cut-off of 1–14/15+ days and their results were similar to those of the main analysis (Table S2).

## Discussion

We examined the joint contribution of combined overweight/obesity and psychological distress with short and long SA periods among young and early midlife Finnish municipal employees. Our study showed that overweight/obesity and psychological distress were associated with increased short and long SA periods and that their joint contribution was strongest among those with overweight/obesity and psychological distress simultaneously than when examined separately. The interaction between overweight/obesity and psychological distress was additive for short SA periods and synergistic for long SA periods. Overall, the joint contribution was stronger with long SA periods than with short SA periods.

To the best of our knowledge, only one prior study has examined the joint contribution of overweight and psychological distress to long-term (≥14 days) SA [19] This study, based on Dutch employees attending occupational health examinations in 2008–12, found a joint contribution among women but no synergistic interaction. In contrast, we observed no significant gender interaction. Differences may reflect variations in study designs, measurements and contexts. The Dutch study relied on self-reported SA, applied gender-stratified analyses, and focused on long-term SA, whereas we included both short- and long-term SA using Employer’s personnel register data. Psychological distress was measured with the Four-Dimensional Symptom Questionnaire (4DSQ) over the past week, while our measure covered a broader timeframe. The studies also differed in occupational sectors, cultural context, and sickness absence practices, which may influence reporting and SA patterns. Given that short SA periods, which are common among young employees [29], predict longer SA periods and long-lasting work disability [34], considering SA of different durations is essential. Moreover, SA episodes were generally more common in our study population, with the majority being short-term, providing a broader perspective on work ability among young employees.

Physiological, socioeconomical, and lifestyle-related factors have been shown to contribute to the bidirectional association between overweight/obesity and psychological distress [35, 36]. Stress activates the hypothalamic-pituitary-axis (HPA), which promotes cortisol secretion and predisposes to abdominal fat accumulation [37]. Overweight/obesity increases the risk of comorbidities, including common mental disorders, which are especially common among young women with obesity [38]. Overweight/obesity and psychological distress tend to overlap and they share at least partly common mechanisms, which might explain their synergistic joint contribution with SA. A stronger joint contribution to longer SA periods might reflect more chronic health problems and long-lasting declines in work ability [31]. The uneven distribution of overweight and psychological distress across socioeconomic layers highlights the importance of addressing the social gradient in health [17]. These conditions often co-occur and may reinforce each other, increasing vulnerability to sickness absence. In our analysis, adjustment for education, physical workload, employment status, and health behaviors did not substantially alter the associations, indicating that the associations between overweight, psychological distress, and SA are robust and extend beyond these explanatory factors. Although unmeasured confounding cannot be excluded, the persistence of these associations emphasizes their relevance.

The joint contribution found with short SA periods suggests targeted interventions could be useful before longer work disability periods. The association with SA was highest among participants with overweight/obesity and psychological distress. Thus, providing support for both health conditions is important as preventive action [36, 39]. It is known that weight management consumes the personal resources of an individual with overweight/obesity [36]. Overcoming weight-related psychological distress, such as stigma and negative self-esteem, enhances an individual’s motivation to initiate and maintain behavioral changes for long-term weight management [36]. For occupational health services and workplaces, these results highlight the importance of programs addressing both mental and physical health. From a societal perspective, reducing sickness absence has implications for productivity, health care costs and overall public health.

### Strengths and limitations

This study had some limitations. First, BMI, psychosocial distress, and all the covariates were self-reported. However, our previous research has confirmed that self-reported BMI has predictive value to SA as effectively as measured BMI, although self-reports are somewhat underreported [40]. The second limitation is that the questionnaire did not directly ask about pregnancy. While we excluded women who reported pregnancy (*n* = 63), some may have gained weight due to pregnancy without mentioning it. However, this is unlikely to have distorted our findings. Third, according to non-response analysis, the non-respondents were more often men, had lower incomes, had part-time jobs, were manual workers and had more SA periods [25]. However, the differences in the distributions of socioeconomic, workplace, and health-related factors between the online, mail, and phone interview respondents were minor and the data satisfactorily represented the target population [25].

A major strength of this study is the large occupational cohort, among the largest employer in Finland, covering hundreds of different occupational titles in the public sector. The comprehensive questionnaire data enabled us to account for several sociodemographic, socioeconomic, lifestyle, and work-related covariates. Another strength was the use of wide-ranging comprehensive register data on SA from employer’s registers. Moreover, the data also covered self-certified, short SA periods (1–7 days) that are seldom studied despite they comprise a majority of SA periods and predict longer SA. Short SA periods are more common among young employees, and they tend to recur more frequently than long-term absences [29]. Including participants with prior SA shorter than 6 months was essential for a representative sample, as short SA are common [29]. Excluding them would introduce bias. We chose not to adjust for prior SA to avoid overadjustment and maintain the integrity of our findings, considering that risk factors like overweight/obesity do not emerge at Phase 1. Short SA affect workplace functioning and the costs arising from short SA fall entirely on the employer and are therefore highly relevant in early-stage preventive occupational health efforts.

As a supplementary analysis, we could also consider different cut-off points for short and long SA periods, but the results remained similar. Finally, to confirm that our joint contributions are not sensitive to the selected cut-off point, we used different cut-off points, 60 points or less, 76 points (median value), and 85 points or more for the emotional wellbeing (RAND-36). Similar results were observed, with associations pointing in the same direction. Differences in the strength of the associations were observed, with associations being stronger when a stricter cut-off point was applied (further results not shown).

## Supplementary Material

ckag092_Supplementary_Data

## Data Availability

The Helsinki Health Study survey data cannot be made publicly available due to strict data protection laws and regulations. The data can only be used for scientific research. More information on the survey data can be requested from the Helsinki Health Study research group (kttl-hhs@helsinki.fi). Key points We examined the joint contribution of overweight/obesity and psychological distress to short (1–7 days) and long (8+ days) sickness absence among young and early midlife Finnish municipal employees. Both conditions were associated with increased sickness absence, with a joint effect, additive for short and synergistic for long absence. This information is useful for employers and occupational health care when planning early interventions for both health issues simultaneously.

## References

[1] World Health Organization. World Health Statistics 2023: Monitoring Health for the SDGs, Sustainable Development Goals. Geneva: WHO, 2023.

[2] Global, regional, and national burden of 12 mental disorders in 204 countries and territories, 1990–2019: a systematic analysis for the global burden of disease study 2019. Lancet Psychiatry 2022;9:137–50. 35026139 10.1016/S2215-0366(21)00395-3 PMC8776563

[3] World Health Organization. European Regional Obesity Report 2022. Copenhagen: WHO Regional Office for Europe, 2022.

[4] Koliaki C , Dalamaga M, Liatis S. Update on the obesity epidemic: after the sudden rise, is the upward trajectory beginning to flatten? Curr Obes Rep 2023;12:514–27. 37779155 10.1007/s13679-023-00527-y PMC10748771

[5] THL. Terve Suomi 2022–23. Helsinki: THL, 2023. https://www.thl.fi/tervesuomi_verkkoraportit/ilmioraportit_2023/lihavuus.html (15 May 2024, date last accessed).

[6] Amiri S , Behnezhad S. Body mass index and risk of sick leave: a systematic review and meta-analysis. Clin Obes 2019;9:e12334. 31368657 10.1111/cob.12334

[7] Pihlajamäki M , Uitti J, Arola H et al Self-reported health problems and obesity predict sickness absence during a 12-month follow-up: a prospective cohort study in 21,608 employees from different industries. BMJ Open 2019;9:e025967. 10.1136/bmjopen-2018-025967 PMC6830705 31676640

[8] Roos E , Laaksonen M, Rahkonen O et al Relative weight and disability retirement: a prospective cohort study. Scand J Work Environ Health 2013;39:259–67. 23060294 10.5271/sjweh.3328

[9] Kelly MJ , Dunstan FD, Lloyd K et al Evaluating cutpoints for the MHI-5 and MCS using the GHQ-12: a comparison of five different methods. BMC Psychiatry 2008;8:10. 18284689 10.1186/1471-244X-8-10 PMC2265280

[10] Aalto AM , Aro A-TJ. *RAND-36 terveyteen liittyvän elämänlaadun mittarina.* 1999. https://urn.fi/URN: NBN: fi-fe201211089642 (18 January 2024, date last accessed).

[11] Parikka S , Holm M, Suvisaari J et al Past trends and future projections of psychological distress among general population in Finland. BMJ Public Health 2025;3:e002026. 40820995 10.1136/bmjph-2024-002026 PMC12352225

[12] European Commission. *Flash Eurobarometer 530: Mental Health*. 2023. https://europa.eu/eurobarometer (16 April 2024, date last accessed).

[13] Halonen JI , Hiilamo A, Butterworth P et al Psychological distress and sickness absence: within- versus between-individual analysis. J Affect Disord 2020;264:333–9. 32056769 10.1016/j.jad.2020.01.006

[14] Terluin B , van Rhenen W, Anema JR et al Psychological symptoms and subsequent sickness absence. Int Arch Occup Environ Health 2011;84:825–37. 21479720 10.1007/s00420-011-0637-4

[15] Kela. *Kela Statistical Yearbook 2023*. 2024. http://hdl.handle.net/10138/588826 (5 May 2025, date last accessed).

[16] Kela. *Kela Statistical Yearbook 2022*. 2023. http://hdl.handle.net/10138/568347 (14 May 2024, date last accessed)

[17] Khanolkar AR , Patalay P. Socioeconomic inequalities in co-morbidity of overweight, obesity and mental ill-health from adolescence to mid-adulthood in two national birth cohort studies. Lancet Reg Health Eur 2021;6:100106. 34308407 10.1016/j.lanepe.2021.100106 PMC8291042

[18] Svärd A , Pipping H, Lahti J et al Joint association of overweight and common mental disorders with diagnosis-specific disability retirement: a follow-up study among female and male employees. J Occup Environ Med 2018;60:979–84. 30020220 10.1097/JOM.0000000000001409

[19] Nigatu YT , Roelen CAM, Reijneveld SA et al Overweight and distress have a joint association with long-term sickness absence among Dutch employees. J Occup Environ Med 2015;57:52–7. 25563539 10.1097/JOM.0000000000000273

[20] Duijts SFA , Kant I, Swaen GMH et al A meta-analysis of observational studies identifies predictors of sickness absence. J Clin Epidemiol 2007;60:1105–15. 17938051 10.1016/j.jclinepi.2007.04.008

[21] de Vries H , Fishta A, Weikert B et al Determinants of sickness absence and return to work among employees with common mental disorders: a scoping review. J Occup Rehabil 2018;28:393–417. 28980107 10.1007/s10926-017-9730-1 PMC6096498

[22] Halonen JI , Lallukka T, Kujanpää T et al The contribution of physical working conditions to sickness absence of varying length among employees with and without common mental disorders. Scand J Public Health 2021;49:141–8. 31960756 10.1177/1403494820901411 PMC7917561

[23] Salonsalmi A , Rahkonen O, Lahelma E et al Changes in alcohol drinking and subsequent sickness absence. Scand J Public Health 2015;43:364–72. 25743874 10.1177/1403494815574154

[24] Troelstra SA , Coenen P, Boot CRL et al Smoking and sickness absence: a systematic review and meta-analysis. Scand J Work Environ Health 2020;46:5–18. 31478055 10.5271/sjweh.3848

[25] Lallukka T , Pietiläinen O, Jäppinen S et al Factors associated with health survey response among young employees: a register-based study using online, mailed and telephone interview data collection methods. BMC Public Health 2020;20:184. 32024488 10.1186/s12889-020-8241-8 PMC7003443

[26] Hays RD , Morales LS. The RAND-36 measure of health-related quality of life. Ann Med 2001;33:350–7. 11491194 10.3109/07853890109002089

[27] OECD. *Measuring Population Mental Health*. 2023. https://www.oecd-ilibrary.org/social-issues-migration-health/measuring-population-mental-health_5171eef8-en (16 April 2024, date last accessed).

[28] Thorsen SV , Rugulies R, Hjarsbech PU et al The predictive value of mental health for long-term sickness absence: the major depression inventory (MDI) and the mental health inventory (MHI-5) compared. BMC Med Res Methodol 2013;13:115. 24040899 10.1186/1471-2288-13-115 PMC3848657

[29] Sumanen H , Pietiläinen O, Lahti J et al Sickness absence among young employees: trends from 2002 to 2013. J Occup Health 2015;57:474–81. 26228519 10.1539/joh.14-0236-OA PMC6706184

[30] Viertiö S , Kiviruusu O, Piirtola M et al Factors contributing to psychological distress in the working population, with a special reference to gender difference. BMC Public Health 2021;21:611. 33781240 10.1186/s12889-021-10560-y PMC8006634

[31] Salmela J , Lahti J, Kanerva N et al Latent classes of unhealthy behaviours and their associations with subsequent sickness absence: a prospective register-linkage study among Finnish young and early midlife employees. BMJ Open 2023;13:e070883. 10.1136/bmjopen-2022-070883 PMC10186467 37169500

[32] Andersson T , Alfredsson L, Källberg H et al Calculating measures of biological interaction. Eur J Epidemiol 2005;20:575–9. 16119429 10.1007/s10654-005-7835-x

[33] OECD. Health at a Glance: Europe 2012. Paris: OECD Publishing, 2012.

[34] Laaksonen M , He L, Pitkäniemi J. The durations of past sickness absences predict future absence episodes. J Occup Environ Med 2013;55:87–92. 23235465 10.1097/JOM.0b013e318270d724

[35] Steptoe A , Frank P. Obesity and psychological distress. Philos Trans R Soc B Biol Sci 2023;378:20220225. 10.1098/rstb.2022.0225 PMC10475872 37661745

[36] Vallis M. Quality of life and psychological well-being in obesity management: improving the odds of success by managing distress. Int J Clin Pract 2016;70:196–205. 26842304 10.1111/ijcp.12765 PMC5067635

[37] Kumar R , Rizvi MR, Saraswat S. Obesity and stress: a contingent paralysis. Int J Prev Med 2022;13:95. 35958362 10.4103/ijpvm.IJPVM_427_20 PMC9362746

[38] Cheng HL , Medlow S, Steinbeck K. The health consequences of obesity in young adulthood. Curr Obes Rep 2016;5:30–7. 26830309 10.1007/s13679-016-0190-2

[39] Durrer Schutz D , Busetto L, Dicker D et al European practical and patient-centred guidelines for adult obesity management in primary care. Obes Facts 2019;12:40–66. 30673677 10.1159/000496183 PMC6465693

[40] Korpela K , Roos E, Lallukka T et al Different measures of body weight as predictors of sickness absence. Scand J Public Health 2013;41:25–31. 23221374 10.1177/1403494812468965

