# Supplementary material for: Joint contribution of body mass index and psychological distress to short and long sickness absence among young and early midlife public sector employees: a register-linked follow-up study
Source: Eur J Public Health. 2026 Jun 10;36(4):ckag092. doi: 10.1093/eurpub/ckag092 (PMC13250733; doi:10.1093/eurpub/ckag092)
Supplement: ckag092_Supplementary_Data [file ckag092_supplementary_data.zip › ejph-2026-01-om-0003-File005.docx]

Supplementary Table S2. Joint associations of overweight/obesity (body mass index ≥25 kg/m^2^) and psychological distress (emotional wellbeing score ≤60) with subsequent sickness absence (SA) periods of 1–14 days and 15+ days among young and early midlife employees of City of Helsinki at Phase 1, 2017 (rate ratios, RRs and their 95% confidence intervals, CIs).

|  |  |  | Model 1^a^ | | Model 2^b^ | | Model 3^c^ | |
| --- | --- | --- | --- | --- | --- | --- | --- | --- |
| SA periods | Exposure group | n (%) | RR | 95% CI | RR | 95% CI | RR | 95% CI |
| 1–14 days | Psychological distress/overweight |  |  |  |  |  |  |  |
|  | Neither | 1,815 (46) | 1.00 |  | 1.00 |  | 1.00 |  |
|  | Psychological distress only | 496 (13) | 1.36 | 1.22–1.52 | 1.30 | 1.16–1.45 | 1.29 | 1.15–1.44 |
|  | Overweight only | 1,230 (31) | 1.21 | 1.12–1.32 | 1.13 | 1.04–1.22 | 1.12 | 1.03–1.21 |
|  | Both | 425 (11) | 1.64 | 1.46–1.84 | 1.47 | 1.31–1.65 | 1.44 | 1.28–1.62 |
| 15+ days | Psychological distress/overweight |  |  |  |  |  |  |  |
|  | Neither | 1,815 (46) | 1.00 |  | 1.00 |  | 1.00 |  |
|  | Psychological distress only | 496 (13) | 1.90 | 1.51–2.38 | 1.75 | 1.39–2.21 | 1.73 | 1.37–2.18 |
|  | Overweight only | 1,230 (31) | 1.34 | 1.11–1.62 | 1.20 | 0.99–1.46 | 1.19 | 0.98–1.44 |
|  | Both | 425 (11) | 3.02 | 2.43–3.75 | 2.60 | 2.08–3.25 | 2.54 | 2.03–3.17 |
| *^a^Model 1: Adjusted for age and gender* | |  |  |  |  |  |  |  |
| *^b^Model 2: Adjusted for age, gender, marital status, education, work status, and physical strenuousness of work* | | | | | | | | |
| *^c^Model 3: Adjusted for age, gender, alcohol consumption, smoking, and leisure-time and commuting physical activity* | | | | | | | | |
