# Supplementary material for: Joint contribution of body mass index and psychological distress to short and long sickness absence among young and early midlife public sector employees: a register-linked follow-up study
Source: Eur J Public Health. 2026 Jun 10;36(4):ckag092. doi: 10.1093/eurpub/ckag092 (PMC13250733; doi:10.1093/eurpub/ckag092)
Supplement: ckag092_Supplementary_Data [file ckag092_supplementary_data.zip › ejph-2026-01-om-0003-File004.docx]

Supplementary Table S1. Joint associations of obesity (body mass index ≥30 kg/m^2^) and psychological distress (emotional wellbeing score ≤60) with subsequent sickness absence (SA) periods of 1–7 days and 8+ days among young and early midlife employees of City of Helsinki, at Phase 1, 2017 (rate ratios, RRs and their 95% confidence intervals, CIs).

|  |  |  | Model 1^a^ | | Model 2^b^ | | Model 3^c^ | |
| --- | --- | --- | --- | --- | --- | --- | --- | --- |
| SA periods | Exposure group | n (%) | RR | 95% CI | RR | 95% CI | RR | 95% CI |
| 1–7 days | Psychological distress/obesity |  |  |  |  |  |  |  |
|  | Neither | 2,641 (67) | 1.00 |  | 1.00 |  | 1.00 |  |
|  | Psychological distress only | 745 (19) | 1.35 | 1.23–1.48 | 1.30 | 1.18–1.42 | 1.28 | 1.17–1.40 |
|  | Obesity only | 404 (10) | 1.43 | 1.28–1.60 | 1.29 | 1.15–1.45 | 1.28 | 1.14–1.44 |
|  | Both | 176 (4) | 1.84 | 1.56–2.17 | 1.60 | 1.35–1.89 | 1.58 | 1.34–1.87 |
| 8+ days | Psychological distress/obesity |  |  |  |  |  |  |  |
|  | Neither | 2,641 (67) | 1.00 |  | 1.00 |  | 1.00 |  |
|  | Psychological distress only | 745 (19) | 1.77 | 1.54–2.04 | 1.70 | 1.48–1.97 | 1.69 | 1.46–1.95 |
|  | Obesity only | 404 (10) | 1.67 | 1.40–1.99 | 1.46 | 1.21–1.74 | 1.44 | 1.20–1.72 |
|  | Both | 176 (4) | 2.69 | 2.14–3.40 | 2.31 | 1.83–2.93 | 2.26 | 1.78–2.87 |
| *^a^Model 1: Adjusted for age and gender* | |  |  |  |  |  |  |  |
| *^b^Model 2: Adjusted for age, gender, marital status, education, work status, and physical strenuousness of work* | | | | | | | | |
| *^c^Model 3: Adjusted for age, gender, alcohol consumption, smoking, and leisure-time and commuting physical activity* | | | | | | | | |
